# Supplementary material for: Magnetocardiography on an isolated animal heart with a room-temperature optically pumped magnetometer
Source: Sci Rep. 2018 Nov 1;8:16218. doi: 10.1038/s41598-018-34535-z (PMC6212485; doi:10.1038/s41598-018-34535-z)
Supplement: Supplementary file 1 — Supplementary Information [file 41598_2018_34535_MOESM1_ESM.pdf]

Supplementary Information: Magnetocardiography on an isolated guinea pig heart using a room-temperature optically pumped magnetometer

Kasper Jensen,[1] Mark Alexander Skarsfeldt,[2] Hans Stærkind,[1] Jens Arnbak,[1]
Mikhail V. Balabas,[1,3] Søren-Peter Olesen,[2] Bo Hjorth Bentzen,[2] and Eugene S. Polzik[1]

[1]Niels Bohr Institute, University of Copenhagen, Blegdamsvej 17, 2100 Copenhagen, Denmark

[2]Department of Biomedical Sciences, Faculty of Health and Medical Sciences, University of Copenhagen, Blegdamsvej 3, 2200 Copenhagen N, Denmark

[3]Department of Physics, St Petersburg State University, Universitetskii pr. 28, 198504 Staryi Peterhof, Russia

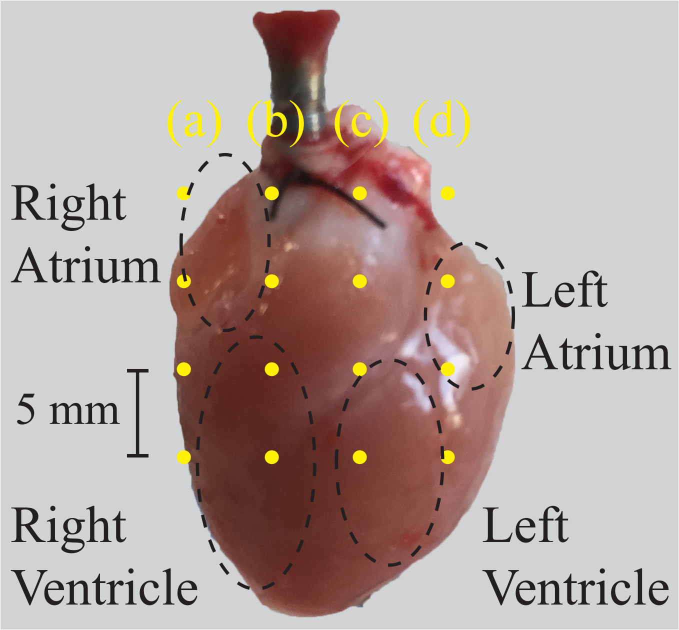

Supplementary Figure S1: Picture of an isolated guinea-pig heart. Approximate locations of Right Atrium, Right Ventricle, Left Atrium, and Left Ventricle are indicated in the figure with dashed ellipses. In the magnetic field measurements shown later in Supplementary Figures 2 and 3, the heart was translated in the y-z-plane such that different regions of the heart were positioned in front of the cesium vapor cell. The approximate relative positions (a), (b), (c), (d), ... are indicated in the figure with yellow dots.

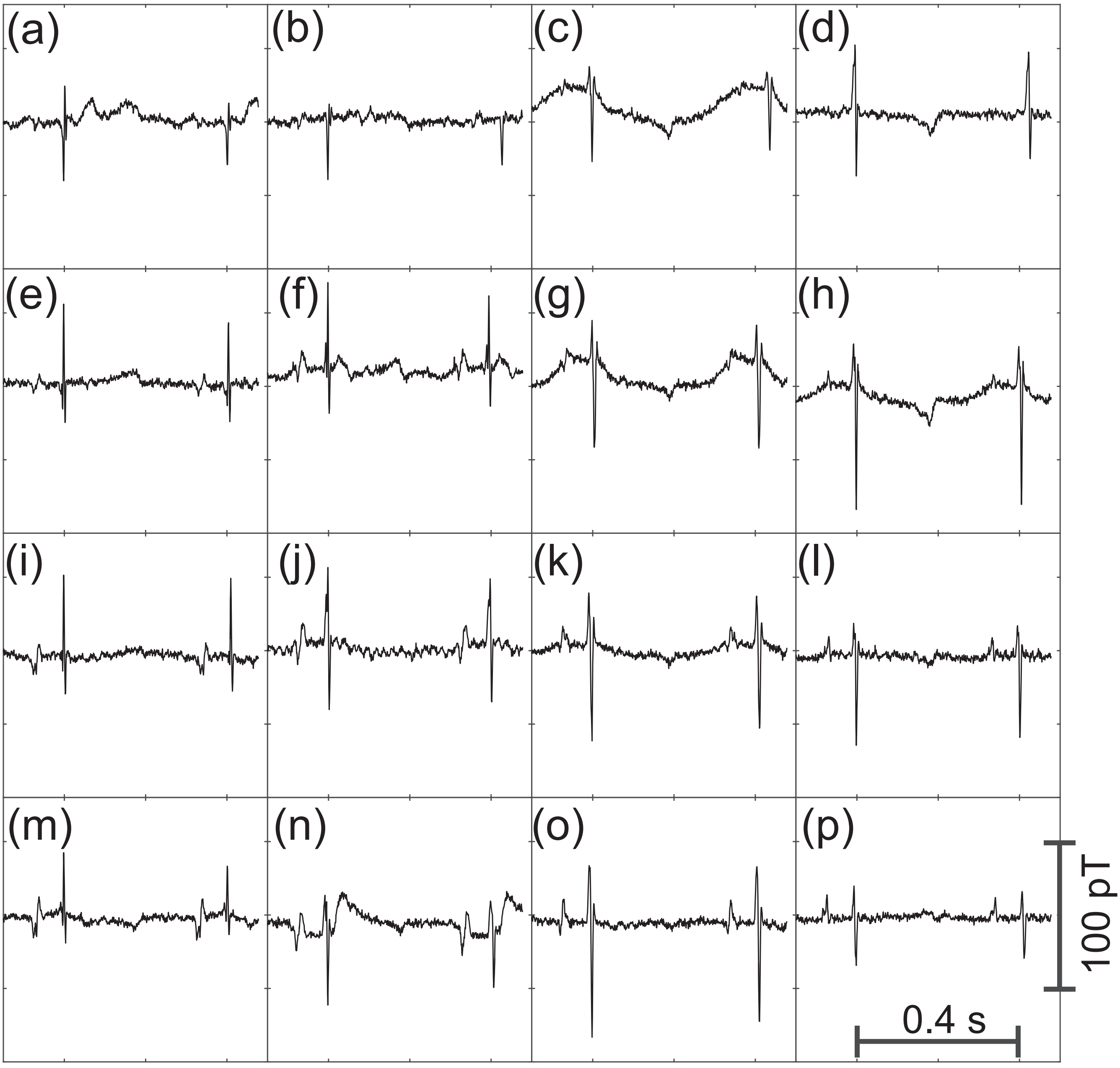

Supplementary Figure S2: Magnetic field from the heart recorded at 16 different positions of the heart relative to the magnetometer (yellow dots in Supplementary Figure S1). In these recordings, the signals were converted to magnetic field units by deconvolution and low-pass filtering using a Type I Chebyshev filter with a cut-off frequency of 500 Hz. For each position, we recorded the magnetic field for 4 seconds. During those 4 seconds, the heart beat approximately 10 times. In the data analysis, the 10 heartbeats were averaged in order to improve the signal-to-noise ratio.

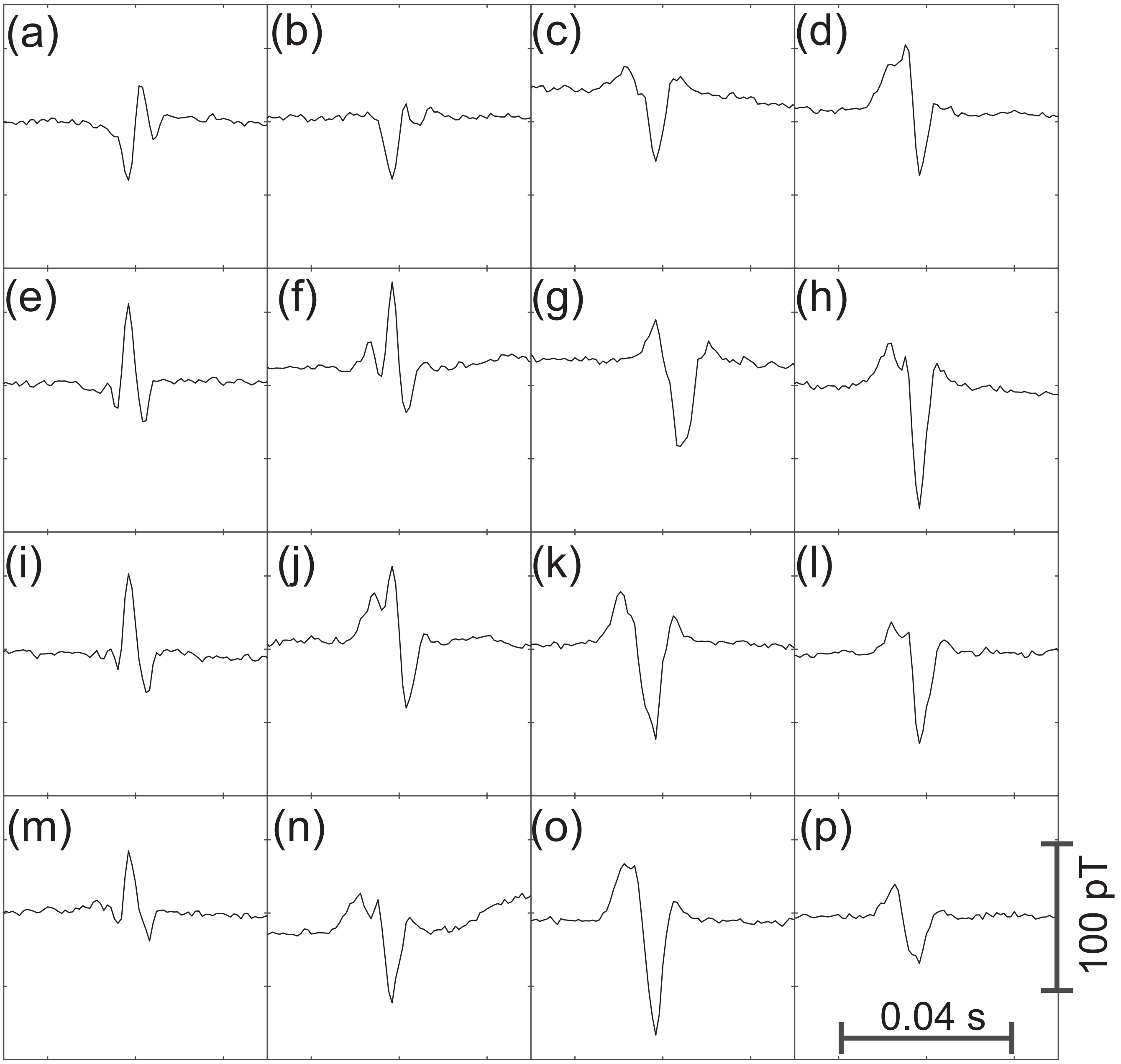

Supplementary Figure S3: Zoom-ins on the QRS-complexes of the magnetocardiograms shown in Supplementary Figure S2. Notice that the morphology and the peak-to-peak amplitude of the QRS-complex depend on the relative position between the heart and the vapor cell.

heart	1	2	3	4	5	6	mean	std
biventricular diameter [mm]	18	14	14	18	17	20	**16.8**	2.4
length [mm]	23	23	24	22	23	25	**23.3**	1.0

Supplementary Table S1: Measured size of six isolated guinea-pig hearts.
